# Anti-Protozoal Activity of Hops Essential Oil and Myrcene Against Cryptosporidium Parvum in Cell Culture

**DOI:** 10.3390/foods14193352

**Published:** 2025-09-27

**Authors:** Danielle F. Aycart, Astrid Domínguez-Uscanga, William H. Witola, Juan E. Andrade Laborde

**Affiliations:** 1 Department of Food Science and Human Nutrition, University of Illinois at Urbana-Champaign, Urbana, IL 61801, USAastrid.dominguez@tec.mx (A.D.-U.); 2 Department of Food Science and Human Nutrition, University of Florida, Gainesville, FL 32611, USA; 3 Facultad de Ciencias de la Vida, Escuela Politécnica del Litoral (ESPOL), Campus Gustavo Galindo Km. 30.5 Vía Perimetral, P.O. Box 09 01 5863, Guayaquil, Ecuador; 4 Tecnológico de Monterrey, Escuela de Ingeniería y Ciencias, Ave. Eugenio Garza Sada 2501, Monterrey 64849, NL, Mexico; 5 Department of Pathobiology, College of Veterinary Medicine, University of Illinois at Urbana-Champaign, Urbana, IL 61802, USA; whwit35@illinois.edu

**Keywords:** *Cryptosporidium parvum*, hops essential oil, myrcene, antiparasitic

## Abstract

Hops essential oil (HEO), a by-product of the brewing industry, has known antibacterial and antifungal properties, but its antiparasitic effects remain underexplored. This study evaluated the cytotoxicity of HEO and its predominant monoterpene, myrcene, in intestinal cells and assessed their ability to reduce *Cryptosporidium parvum* infection in vitro. The cytotoxicity (IC_50_) of HEO and myrcene was determined in HCT-8 intestinal cells using flow cytometry and propidium iodide staining after 24 and 48 h of exposure. The anti-*Cryptosporidium* activity of HEO and myrcene was assessed by infecting confluent HCT-8 cells with *C. parvum* sporozoites (1 × 10^4^ sporozoites/mL) and treating them with bioactives below their IC_50_ values. Two treatment modalities were tested: (1) immediate treatment during infection (invasion) and (2) treatment initiated 2 h after infection (growth). Parasite growth was quantified using an immunofluorescence assay with a fluorescence-conjugated anti-*Cryptosporidium* antibody. HEO exhibited low cytotoxicity (IC_50_ = 382.7 µg/mL), while myrcene showed higher cytotoxicity (IC_50_ = 240.6 µg/mL). HEO reduced *C. parvum* growth in a dose-dependent manner, with IC_50_ values of 45.8 and 58.7 µg/mL under either modality, respectively. Myrcene alone demonstrated greater anti-*Cryptosporidium* activity, with IC_50_ values lower under the invasion modality (17.7 µg/mL) than the growth modality (28.1 µg/mL) on average for both food-grade and analytical standards. HEO and myrcene exhibited significant in vitro anti-*Cryptosporidium* activity, highlighting their potential as novel therapeutic agents against cryptosporidiosis.

## 1. Introduction

Diarrheal disease is one of the top five causes of death in children under five years old, according to the World Health Organization [1]. It can be caused by various bacterial, viral, and parasitic organisms [2]. Among the parasites responsible for significant morbidity and mortality in children are helminths including *Ascaris lumbricoides* (roundworm), *Trichuris trichiura* (whipworm), *Ancylostoma duodenale*, and *Necator americanus* (hookworms) [3]. Protozoan parasites such as *Giardia intestinalis*, *Entamoeba histolytica*, and *Cryptosporidium* spp. also contribute to diarrheal disease [4]. The two primary *Cryptosporidium* species infecting humans are *Cryptosporidium parvum* and *Cryptosporidium hominis* [5].

*C. parvum* infection occurs via the fecal-oral route, typically through the ingestion of oocysts. Once inside the host, *C. parvum* infects intestinal cells and undergoes both asexual and sexual reproduction, leading to further intestinal cell invasion and oocyst shedding through feces to continue its life cycle [4]. Due to their thick-walled membrane, oocysts are highly resistant to harsh environmental conditions and common chemical disinfection methods such as chlorination. In addition to their resistance to disinfection, *C. parvum* oocysts can persist for months in soil, water, and food, making contaminated drinking water and foods common sources of infection [6]. This environmental stability contributes to waterborne outbreaks, particularly in regions with inadequate sanitation infrastructure. Consequently, populations in low-income countries with limited access to clean water are at an increased risk of infection. The consequences of *C. parvum* infection extend beyond diarrhea and are influenced by the host’s age, nutritional status, and immune function [7]. Malnourished children under five are particularly vulnerable, experiencing long-term cognitive deficits, growth impairments, and increased susceptibility to secondary infections [8].

Various drugs have demonstrated anti-*Cryptosporidium* activity in in vitro, in vivo, and clinical studies [6]. Nitazoxanide is currently the only FDA-approved treatment for cryptosporidiosis; however, its efficacy is significantly reduced in malnourished children [6,9,10]. This limitation has led to a growing interest in developing novel therapeutics to combat *Cryptosporidium* infection [11]. Plant-derived bioactives have emerged as potential alternatives due to their natural antimicrobial and antiparasitic properties [12]. Several plant-derived compounds, including garlic extracts (allicin) [13], chicory root (*Cichorium intybus*) [14], and curcumin (*Curcuma oblonga*) [15], have shown promising anti-*Cryptosporidium* activity in experimental models.

The brewing industry generates substantial quantities of plant-based by-products, of which hops essential oil (HEO) represents a promising circular ingredient for its known anti-microbial properties. HEO is typically obtained during hops resin extraction, especially via steam distillation, supercritical CO_2_ extraction, hydrodistillation of hops pellets before or even after dry hopping, a process integral to beer flavoring, and is often discarded or underutilized despite its high bioactive content [16,17]. Incorporating HEO as a functional ingredient aligns with global goals of waste valorization and circular bioeconomy strategies aimed at reducing industrial waste streams while promoting health-oriented applications [18]. HEO has received significant attention for its application in drugs, pesticides, and functional foods [19,20]. It is primarily composed of monoterpenes and sesquiterpenes, with myrcene being the most abundant monoterpene along with the oxygenated form linalool, comprising 30–60% of the total oil. Humulene, caryophyllene, and farnesene constitute the remaining sesquiterpenes [21]. Traditionally, hops have been used for food preservation and crop protection due to their antimicrobial properties [21,22,23]. Previous evidence has demonstrated the antifungal [24], antibacterial [25], and anti-parasitic activity [26] of essential oils containing myrcene. Hops essential oil and myrcene have shown anti-protozoan action against *Toxoplasma gondii* and *Eimeria tenella* [27], as well as *Tripanosama brucei* [28]. The antimicrobial efficacy of HEO is largely attributed to its ability to disrupt microbial membranes, inhibit oxidative processes, and interfere with essential metabolic pathways [29,30]. Given these mechanisms, it is plausible that HEO bioactives, particularly myrcene and sesquiterpenes, could impair *Cryptosporidium* development and survival within host cells. However, limited research has explored the antiparasitic properties of HEO and myrcene against *Cryptosporidium* spp. This study evaluated the effect of HEO and myrcene on *C. parvum* infection in HCT-8 cells in culture.

## 2. Materials and Methods

### 2.1. Materials and Reagents

The following reagents were obtained from Thermo-Fisher Scientific (Waltham, MA, USA): TrypLE™ Express Enzyme (1X) without Phenol Red, CountBright™ Absolute Counting Beads, Dimethylsulfoxide (DMSO), and Trypan blue solution (0.4%). RPMI 1640 culture media with L-glutamine and Phenol Red was obtained from GibcoTM (Grand Island, NY, USA) and supplemented with 10% Horse Serum, 2 g/L of sodium bicarbonate, 2.5 g/L of glucose, 1× antibiotic–antimycotic (GibcoTM), and 1× sodium pyruvate (GibcoTM). Human ileocecal colorectal adenocarcinoma cells (HCT-8) obtained from American Type Culture Collection (ATCC^®^, CCL-244, Manassas, VA, USA). *C. parvum* oocysts extracted from fresh feces of an infected male Holstein calf were kindly provided by Dr. William Witola (Department of Pathobiology, University of Illinois at Urbana-Champaign, Urbana, IL, USA).

Hops (*Humulus lupulus*) essential oil (HEO) was donated by John Hass (Barth-Hass Group, Yakima, WA, USA) containing 62% myrcene (as β-myrcene with a relative density: 0.846 g/mL at 23 °C). Food-Grade Myrcene (FGM) (purity: ≥90%, relative density: 0.790 g/mL at 21 °C, molar absorptivity: 15,350 M^−1^.cm^−1^) was purchased from Elevation Terpenes (Goleta, CA, USA). Analytic grade standard myrcene (AGM) (purity: ≥90%, relative density: 0.791 g/mL at 25 °C) was purchased from Sigma-Aldrich (Cat.No. 64643, St. Louis, MO, USA).

### 2.2. Cytotoxicity Evaluation

The in vitro cytotoxicity of HEO and myrcene was evaluated in HCT-8 cells to establish a range of non-cytotoxic concentrations to be tested against *C. parvum*. Cell cytotoxicity of HEO and myrcene was determined by flow cytometry. HCT-8 cells were seeded, maintained, and treated in T-25 flasks (Thermo Fisher Scientific, Whatman, MA, USA) containing 5 mL of RPMI 1640 medium and incubated at 37 °C, 5% CO_2_ atmosphere. HEO and myrcene were tested at final concentrations of 0, 100, 300, 500, 700, and 1000 µg/mL to determine the dose–response relationship and calculate IC_50_ values. The compounds were first dissolved in DMSO, then further diluted in complete RPMI 1640 medium, and tested in triplicate. The DMSO concentration in all treatments did not exceed 0.1%. Cells were cultured to 80–90% confluence before treatment, followed by incubation at 37 °C for either 24 or 48 h. A negative control was included, consisting of a culture medium supplemented with 0.1% DMSO. Following treatment, cells were detached using TrypLE™ Express, neutralized with medium, centrifuged at 800 rpm for 5 min, and resuspended in fresh RPMI medium at a density of 10^6^ cells/mL. Samples were then analyzed using a BD LSR II Flow Cytometry Analyzer (BD Biosciences, San Jose, CA, USA). Prior to analysis, cells were stained with 2 μg/mL propidium iodide (488 nm) for 5 min in the dark, then briefly vortexed before placement in the flow cytometer. Dot plots of forward scatter area (FSC-A) versus side scatter area (SSC-A) were generated using FACSDiVa™ v6.1 software. Voltages were optimized to properly gate single-cell populations, ensuring the exclusion of debris. Cells expressing no fluorescence were classified as live, while those in the lower right region were categorized as dead. The half-maximal inhibitory concentrations (cytotoxicity IC_50_ values) were determined by non-linear regression using GraphPad Prism^®^ version 5.0 (GraphPad Software Inc., La Jolla, CA, USA).

### 2.3. C. parvum Preparation

Sporozoites were excysted from *C. parvum* oocysts following the procedure outlined by Kuhlenschmidt et al. (2016) [31]. The initial concentration of the oocyst stock (3.3 × 10^8^ oocysts/mL) was diluted to 1 × 10^8^ purified oocysts with 500 μL PBS, and an equal volume of 40% commercial bleach. The mixture was incubated for 10 min at 4 °C with occasional inverting to facilitate surface decontamination. To rinse, oocysts were first centrifuged at 13,200 rpm (16,000× *g*) for 1 min at 23 °C (5415-D, Eppendorf, Hamburg, Germany). Then, the pellet was rinsed with 1 mL of 1% (*w*/*v*) BSA in PBS. Oocysts were rinsed four times. After the final rinse, oocysts were suspended in Hanks’ balanced salt solution (HBSS). To induce excystation, the suspension was incubated at 37 °C for 60 min, then combined with an equal volume of warm 1.5% sodium taurocholate in HBSS and incubated for an additional 60 min at 37 °C. The excysted sporozoites were then collected by centrifugation, resuspended in RPMI 1640 medium, and purified by passing the suspension through a sterile 5.0 μM syringe filter (Millex-SV, MilliporeSigma, Burlington, MA, USA). Sporozoites were counted using a hemocytometer and maintained on ice until use.

### 2.4. In Vitro Anti-Cryptosporidium Activity

To assess the efficacy of HEO and myrcene against *C. parvum* infectivity, concentrations below their IC_50_ cytotoxicity values in HCT-8 cells were used. The compounds were first dissolved in 0.1% DMSO and then diluted in RPMI 1640 medium. For these assays, efficacy was evaluated under conditions that reflected parasite invasion (modality 1) or growth (modality 2). For the parasite invasion assay, HCT-8 cell monolayers were cultured in 96-well plates and pre-treated with 200 μL of HEO or myrcene at varying concentrations (0, 30, 50, 70, and 100 μg/mL) immediately before infection with *C. parvum* sporozoites (4 × 10^4^ sporozoites/well). Cells were incubated for 48 h (37 °C and 5% CO_2_) to determine whether the compounds could inhibit sporozoite invasion. For the parasite growth assay, HCT-8 monolayers were first infected with *C. parvum* sporozoites (4 × 10^4^ sporozoites/well) in 200 μL of RPMI 1640 medium and incubated for 2 h. Following infection, the medium was carefully replaced with 200 μL of fresh RPMI 1640 medium containing different concentrations of HEO or myrcene (0, 30, 50, 70, and 100 μg/mL) for an additional 46 h. In both modalities, AGM addition at 100 µg/mL was not evaluated. In all experiments, negative control wells were treated with 0.1% DMSO for the same duration as the treatment groups. Paromomycin was included as a reference drug control in both types of experimental modalities, with HCT-8 monolayers incubated for 48 h with varying concentrations (0, 50, 100, 200, 400, and 500 μg/mL).

### 2.5. Immunofluorescence Determination of C. parvum Growth

Cells were prepared for immunofluorescence analysis following a previously described protocol [31]. After removing the culture medium, cells were fixed with methanol-acetic acid (9:1 *v*/*v*) for 5 min at room temperature. Permeabilization was achieved through successive washes using a buffer solution containing 0.1% Triton X-100, 0.35 M NaCl, and 0.13 M Tris-base (pH 7.6). To minimize nonspecific binding, cells were blocked with 5% normal goat serum, followed by overnight incubation at 4 °C with a fluorescein (FL)-labeled anti-*C. parvum* antibody (SporoGlo; Waterborne, Inc., New Orleans, LA, USA). Following antibody staining, cells were washed twice in PBS, and 200 μL of water was added to each well before imaging. Fluorescence microscopy was performed using a 20× objective on an inverted fluorescence microscope, and quantification of parasite fluorescence in the captured images was conducted using ImageJ software (version 1.37v, National Institutes of Health, Bethesda, MD, USA).

### 2.6. Statistical Analyses

Results are reported as means ± SD. The cytotoxicity of HEO and myrcene was evaluated in triplicate. Data analyses of the images were performed in BD FACSDiVa™ v6.1 and the IC_50_ was obtained after plotting data points (% inhibition) using GraphPad Prism v8 (GraphPad Software Inc., La Jolla, CA, USA). The *C. parvum* invasion and growth assays were evaluated in triplicates and the mean fluorescence intensity was obtained. Selectivity Index (SI) was calculated to assess the safety margin between cytotoxic and antiparasitic concentrations. SI was defined as the ratio of the compound’s IC_50_ for cytotoxicity in HCT-8 cells to its IC_50_ for *C. parvum* inhibition in either invasion or growth assays. A higher SI indicates greater parasite-specific activity relative to host cell toxicity. Data curation was performed in MS Excel and statistical analyses were performed using GraphPad Prism. Comparison between treated and untreated samples was tested using one-way ANOVA, and differences were considered significant at α < 0.05. Conditions for ANOVA such as normal distribution and homoscedasticity were evaluated. After ANOVA, post hoc comparisons among treatments were performed using Tukey–Kramer test, and differences were considered significant at α < 0.05.

## 3. Results

### 3.1. Evaluation of HEO and Myrcene Cytotoxicity

**Figure 1:**
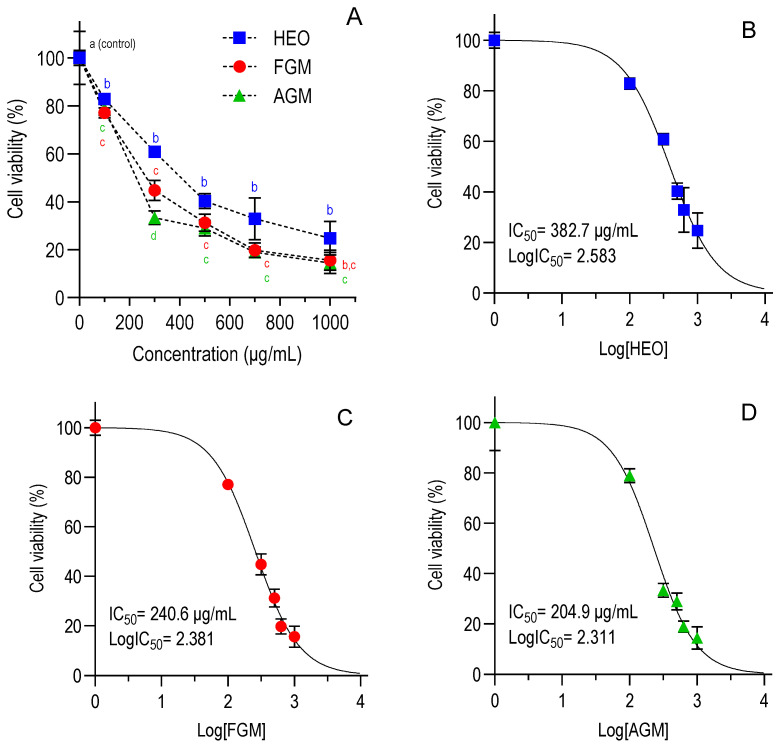
Effect of HEO, FGM, and AGM on HCT-8 cell viability after 48 h of exposure. (**A**) Combined dose–response curves. (**B**–**D**) non-linear sigmoidal dose–response curves for HEO (**B**), FGM (**C**), and AGM (**D**), respectively. Data points represent means ± SD from duplicates across three independent trials. Half-maximal inhibitory concentrations (IC_50_) were determined using non-linear regression after normalization in GraphPad Prism v10.4.1. Different colored superscript letters (a–d) above each concentration point in panel A indicate significant differences among the untreated control and the three treatments at the same concentration and among themselves (*p* < 0.05, one-way ANOVA with Tukey’s *post hoc* test). Points sharing the same letter within a given concentration are not significantly different. No statistical comparisons were made between different concentration levels. Hops essential oil (HEO), food-grade myrcene (FGM), analytical-grade myrcene (AGM), and control (dimethyl sulfoxide, DMSO).

The cytotoxic effect of HEO and myrcene, obtained from two sources, in HCT-8 cells was evaluated by flow cytometry using propidium iodide staining (Figure S1). Dose–response curves for the compounds after 48 h of treatment of the cell cultures were derived (Figure 1). Table 1 shows the half-maximal inhibitory concentration (IC_50_) values for the compounds under evaluation. All treatments elicited a dose-dependent decrease in the viability of the cells with IC_50_ values decreasing with a longer period of exposure (*p* < 0.05). HEO was the least cytotoxic at both times. Purified myrcene, food grade or analytical standard, showed similar cytotoxicity, with lower IC_50_ values at 48 h compared to HEO. All compounds significantly reduced cell viability (50%) at concentrations above 200 µg/mL, confirmed by flow cytometry analyses (Figure S1).

### 3.2. In Vitro Anti-Cryptosporidium Assay

**Figure 2:**
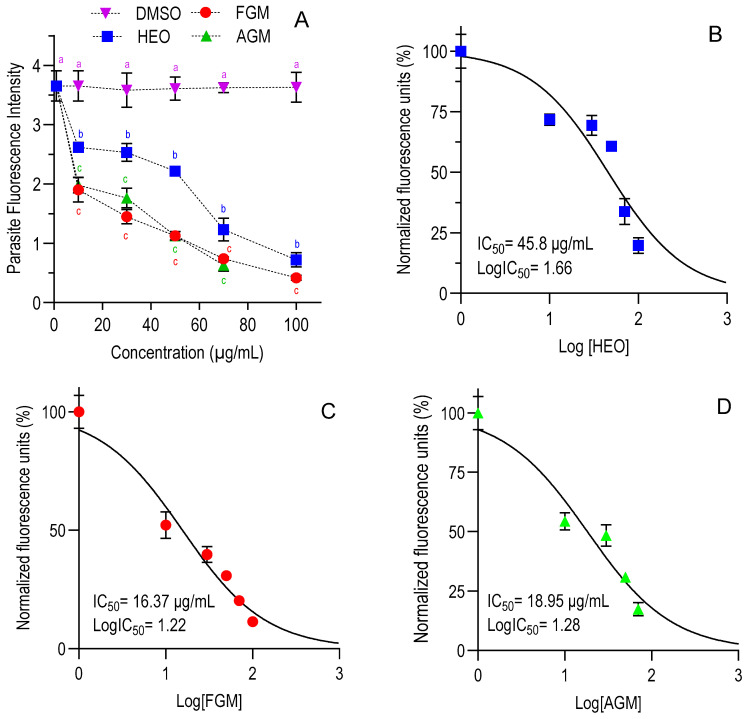
Dose-dependent effect of HEO, FGM, and AGM on relative *C. parvum* growth under the invasion modality. (**A**) Dose–response curves. (**B**–**D**) Non-linear sigmoidal dose–response curves for HEO (**B**), FGM (**C**), and AGM (**D**), respectively. Data points represent means ± SD from duplicates across three independent trials. Half-maximal inhibitory concentrations (IC_50_) were determined using non-linear regression after normalization in GraphPad Prism v10.4.1. Different colored superscript letters (a–c) above each concentration point in panel A indicate significant differences among the untreated control and the three treatments at the same concentration and among themselves (*p* < 0.05, one-way ANOVA with Tukey’s *post hoc* test). Points sharing the same letter within a given concentration are not significantly different. No statistical comparisons were made between different concentration levels. Hops essential oil (HEO), food-grade myrcene (FGM), analytical-grade myrcene (AGM), and control (dimethyl sulfoxide, DMSO).

**Figure 3:**
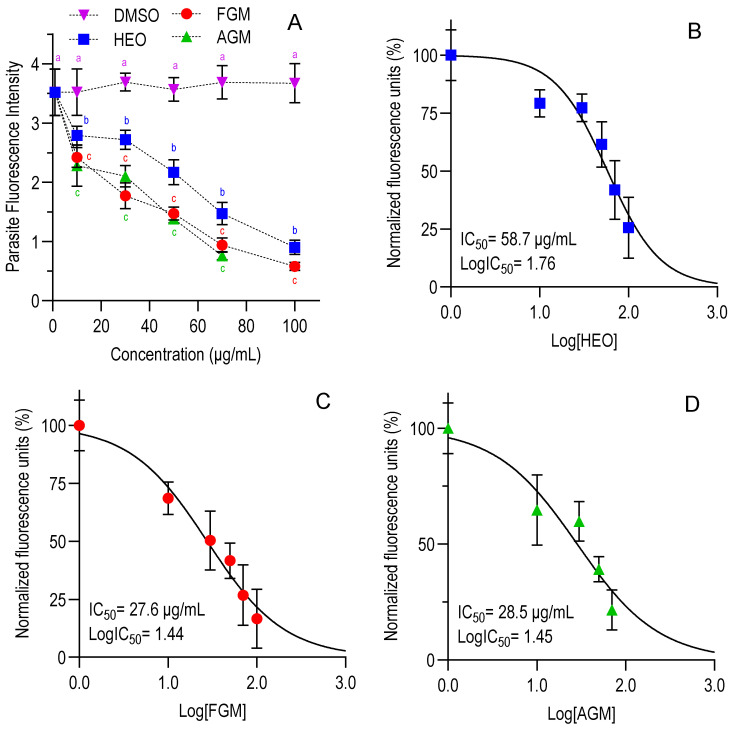
Dose-dependent effect of HEO, FGM, and AGM on relative *C. parvum* growth under the growth modality. (**A**) Dose–response curves. (**B**–**D**) Non-linear sigmoidal dose–response curves for HEO (**B**), FGM (**C**), and AGM (**D**), respectively. Data points represent means ± SD from duplicates across three independent trials. Half-maximal inhibitory concentrations (IC_50_) were determined using non-linear regression after normalization in GraphPad Prism v10.4.1. Different colored superscript letters (a–c) above each concentration point in panel A indicate significant differences among the untreated control and the three treatments at the same concentration and among themselves (*p* < 0.05, one-way ANOVA with Tukey’s *post hoc* test). Points sharing the same letter within a given concentration are not significantly different. No statistical comparisons were made between different concentration levels. Hops essential oil (HEO), food-grade myrcene (FGM), analytical-grade myrcene (AGM), and control (dimethyl sulfoxide, DMSO).

Once the non-cytotoxic doses of HEO and myrcene were established, a range of lower concentrations (0–100 μg/mL) of each compound was evaluated against *C. parvum* invasion (modality 1) and growth (modality 2) in HCT-8 cells (Figure S2). Regardless of the assay modality, all compounds inhibited both the invasion and growth of *C. parvum* in a dose-dependent manner (Figure 2A and Figure 3A). As shown in Table 1, the IC_50_ values for HEO under the two assay modalities were similar (*p* > 0.05), indicating comparable efficacy in preventing parasite invasion and intracellular growth. However, for both assay modalities, HEO exhibited a significantly lower inhibitory effect than myrcene (*p* < 0.05), while the two myrcene forms tested demonstrated similar efficacy (*p* > 0.05). The IC_50_ of paromomycin against *C. parvum* was, on average, for both modalities 106 μg/mL (Figure S3).

## 4. Discussion

The present study is among the first to demonstrate the antiparasitic potential of hops essential oil (HEO) and its major monoterpene, myrcene, against *Cryptosporidium parvum*. While HEO has been previously recognized for its antibacterial and antifungal properties [24,25,26], its efficacy against protozoan parasites has not been established. Both HEO and myrcene significantly inhibited *C. parvum* growth in vitro at concentrations well below the control drug paromomycin and their cytotoxic thresholds in human intestinal epithelial cells (HCT-8), suggesting a favorable therapeutic index.

Previous studies on HEO and its constituent terpenes, including myrcene, have primarily focused on their cytotoxic and antiproliferative effects in cancer cell models. Myrcene, for example, has demonstrated cytotoxicity in HeLa cells [32], MCF-7 breast carcinoma [33], A549 lung adenocarcinoma cells [34], oral squamous carcinoma (SCC-9) cells [35] and other tumor cell lines [36], with IC_50_ values as low as 0.5 µg/mL, depending on cell type and assay conditions [34,36]. These effects are often attributed to membrane-disruptive properties, mitochondria electron transport disruption, oxidative stress induction, and interference with cell cycle progression. However, data on myrcene’s safety in non-transformed intestinal cells—especially absorptive epithelial models relevant to parasitic infection—remain scarce. In the current study, HEO and myrcene exhibited moderate cytotoxicity in HCT-8 cells, with IC_50_ values of 382.7 µg/mL and 240.6 µg/mL, respectively. Despite similar nominal purity, subtle differences in minor constituents, isomeric composition, or residual solvents could influence cytotoxicity. In addition, cytotoxicity can depend on physicochemical properties such as lipophilicity, partitioning into cell membranes, and micelle formation, which affect cellular uptake [37]. We did not characterize particle size distribution or detailed solubility parameters beyond verifying that all compounds dissolved completely and remained visually clear in DMSO under our experimental conditions. These unmeasured factors may explain the small but measurable difference in host–cell viability observed between FGM and AGM. The IC_50_ values, which were derived from non-linear regression of the evaluated concentration–response data, are considerably higher than the concentrations required for antiparasitic activity, suggesting a sufficient therapeutic window for further investigation. When compared to cytotoxicity profiles of other monoterpenes such as limonene and linalool, which also display low micromolar IC_50_ values in various cancer cell lines (linalool, 0.06 μg/mL in HepG2 cells [38]; limonene, 0.55 μg/mL in HepG2 cells [39]), our findings underline the importance of cell-type specificity and reinforce the potential of HEO and myrcene as safe, gut-targeted agents when used at sub-cytotoxic concentrations.

Intestinal protozoan parasites such as *C. parvum*, *Giardia lamblia*, and *Entamoeba histolytica* cause significant human disease, often manifesting as diarrheal illness. Treatment options are limited (e.g., nitazoxanide for cryptosporidiosis, metronidazole for giardiasis and amoebiasis), and issues of suboptimal efficacy or drug resistance spur interest in alternative therapies [4,5]. Essential oils (volatile plant extracts) and their constituents, e.g., the monoterpenes myrcene, thymol, carvacrol, and limonene, have shown broad antimicrobial and antiparasitic properties [40,41,42]. In this study, HEO and myrcene exhibited dose-dependent inhibitory effects against *C. parvum* invasion and growth in the cell culture model; however, the isolated compounds showed greater inhibitory activity than the essential oil. This might be due to the relatively lower concentration of myrcene in the HEO (~62%) and the presence of other sesquiterpenes such as α-humulene and β-caryophyllene, which might have weaker anti-*C. parvum* activity. While these sesquiterpenes have shown antiparasitic effects against *Schistosoma mansoni* [43], their efficacy against *C. parvum* appears limited.

Mono and sesquiterpenes appear to act via multiple mechanisms, both direct (pharmacological effects on the parasite) and indirect (immunomodulatory effects on the host) [44]. Hydrophobic essential oil components can act at the intestinal site of infection. Monoterpenes like myrcene, linalool, and limonene are highly lipophilic and volatile, which enables them to partition into cell membranes and cross biological barriers, both of the host and invaders [45]. When administered orally, these compounds may exert local effects in the gut lumen before significant absorption and metabolism occur. This is ideal for luminal parasites. For example, *Giardia* trophozoites (which reside in the small-intestinal lumen) are directly exposed to ingested essential oils. Even for parasites that invade epithelial cells like *Cryptosporidium*, essential oil constituents in the intestinal lumen can diffuse into the gut lining and reach parasites at the brush-border surface. In this study, the inhibitory effects of HEO were similar between modalities. When given alone, myrcene inhibited *C. parvum* more efficiently during parasite invasion than during intracellular growth (FGM IC_50_ only: 16.4 vs. 27.6 µg/mL). The lower IC_50_ in invasion vs. growth assays suggests that the compound is more effective at stopping the parasite before it enters host cells (invasion). Once the parasite has invaded and started growing, a higher concentration is needed to inhibit further development. This implies that the timing of treatment matters, in which early intervention could be more effective at lower doses.

Though evidence from pharmacokinetic studies is limited in humans, one study showed that when myrcene was given in a 1 mL extract from mastiha oil, a resin from *Pistacia lentiscus* (containing 8.5% myrcene), to healthy volunteers resulted in a large AUC (i.e., larger that the main terpene, α-pinene at 84%), which started 30 min after ingestion and a Cmax (966.6 ± 89.7 μg/L) after 2 h [46]. The reported peak concentrations are typically in the sub-microgram to ~1 µg/mL range, which is well below the in vitro IC_50_ values observed for growth inhibition. Comparable findings have been reported for other hop terpenes and prenylflavonoids, which exhibit low systemic exposure due to high volatility, rapid first-pass metabolism, and efficient clearance [36,47]. These characteristics suggest that achieving equivalent serum concentrations through conventional oral dosing may be challenging. However, *C. parvum* primarily infects the intestinal epithelium, where orally delivered essential oils can attain much higher local concentrations before absorption and metabolism dilute them. This supports a therapeutic strategy focused on local gut action rather than systemic delivery. Furthermore, encapsulation or other gastro-retentive formulations, as we and others have shown [48,49,50], could help preserve these volatile compounds during gastric transit, enhance mucosal contact, and prolong their luminal residence time, thereby maximizing antiparasitic efficacy at sub-cytotoxic doses.

Essential oils are complex mixtures, but their antiparasitic effects can be distilled into a few key mechanisms at the parasite level. These include disruption of parasite cell membranes and organelles; interference with parasite metabolic processes and enzymes; and induction of parasite cell death pathways or developmental arrest [44]. In the case of *Cryptosporidium*, there is limited literature on the mechanisms of action of essential oils. A study by Tanghort et al. (2019) evaluated the oocysticidal activity of thymol and carvacrol against *C. baileyi* and *C. galli* [51]. The researchers observed that treatment with these compounds led to the release of substances absorbing at 273 nm, indicative of membrane damage and oocyst lysis in a dose- and time-dependent manner. The evaluation of essential oils on other protozoan parasites supports this mechanism of action. For instance, Machado et al., demonstrated that essential oils from clove (*Syzygium aromaticum*) and its major compound, eugenol, can compromise the membrane integrity of *Giardia* trophozoites, leading to cell death [52]. Also, neo-clerodane diterpenes from chia (*Salvia polystachya*) cause morphological changes and membrane damage in *E. histolytica*, resulting in reduced viability [53]. Similarly, ursolic acid [54] and geraniol [55] elicited morphological and ultrastructural alteration of trophozoites of *T. vaginalis*, disrupting its cellular functions. These findings support the hypothesis that terpenes such as myrcene can cross and disrupt membranes, and thus, interfere with the early stages of parasite development, including epithelial invasion and intracellular survival.

## 5. Conclusions

This study demonstrated that HEO and myrcene, circular products from the beer industry, effectively inhibit *C. parvum* infection in vitro while maintaining low cytotoxicity in HCT-8 cells. HEO and myrcene exhibited dose-dependent antiparasitic activity at concentrations below their IC_50_ values, highlighting their potential as novel therapeutic alternatives. Further work should evaluate the direct interaction of myrcene with parasite membranes using imaging or membrane potential assays. Additionally, exploring the antiparasitic effects of other oxygenated monoterpenes and sesquiterpenes present in HEO, such as humulene and caryophyllene, could provide insight into potential synergistic or antagonistic interactions.

## Figures and Tables

**Table 1:** IC_50_ values (μg/mL) with 95% confidence intervals [CI 95%] for hops essential oil and myrcene against HCT-8 cell viability (24 and 48 h) and *C. parvum* invasion or growth in vitro.

	Cell Viability		Anti-Cryptosporidium Activity		
	IC_50_ 24 h [CI 95%]	IC_50_ 48 h [CI 95%]	IC_50_ Invasion [CI 95%]	IC_50_ Growth [CI 95%]	Selectivity Index
HEO	675.2 [551.7–829.6]	382.7 [338.9–431.7]	45.8 [35.6–58.8]	58.7 [46.4–72.9]	6.5–8.4
FGM	980.9 [823.0–1178]	240.6 [210.1–274.7]	16.4 [13.3–19.1]	27.6 [19.9–36.3]	8.7–14.7
AGM	506.6 [434.9–590.1]	204.9 [166.7–249.7]	19.0 [14.6–24.3]	28.5 [19.0–40.6]	7.2–10.8

## Data Availability

The data generated and analyzed during the present study are in the domain of the corresponding author and will be made available upon request.

## Supplementary Materials

The following supporting information can be downloaded at: https://www.mdpi.com/article/10.3390/foods14193352/s1. Figure S1. Flow cytometry apoptosis representative images of HCT-8 cells after 48 h of exposure with several concentrations of HEO, FGM and AGM. Hops Essential oil (HEO), Food grade myrcene (FGM), and analytical grade myrcene (AGM); Figure S2. Representative images of HCT-8 cell monolayers after treatment with HEO, FGM, or AGM. All images were captured at 40× magnification; no panel is shown for AGM at 100 μg/mL because this concentration was not tested; Figure S3. Non-linear sigmoidal dose-response curves for paromomycin in the two experimental modalities. Data points represent means ± SD from duplicates across three independent trials. Half-maximal inhibitory concentrations (IC50) were determined using nonlinear regression after normalization in GraphPad Prism v10.4.1.
