# Supplementary material for: Anti-Protozoal Activity of Hops Essential Oil and Myrcene Against Cryptosporidium Parvum in Cell Culture
Source: Foods. 2025 Sep 27;14(19):3352. doi: 10.3390/foods14193352 (PMC12523311; doi:10.3390/foods14193352)

Supplementary Figures

Figure S1. Flow cytometry apoptosis representative images of HCT-8 cells after 48 h of exposure with several concentrations of HEO, FGM and AGM. Hops Essential oil (HEO), Food grade myrcene (FGM), and analytical grade myrcene (AGM).

Concentration μg/mL	HEO	FGM	AGM
1000	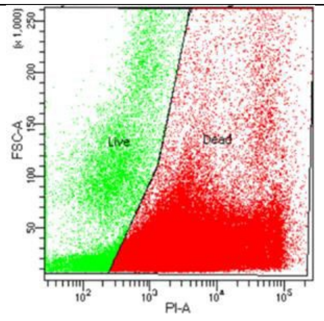	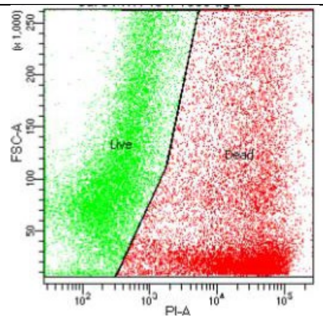	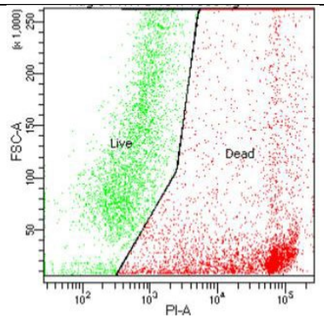
700	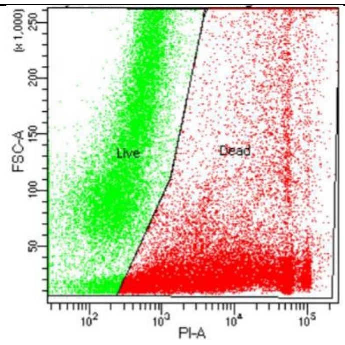	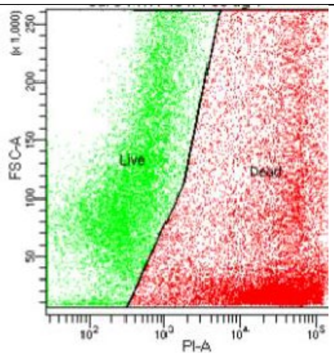	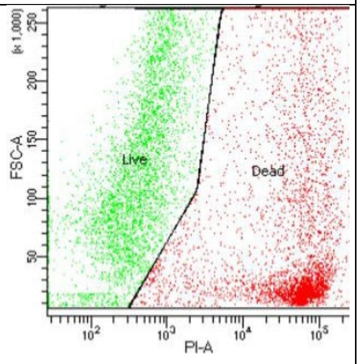
500	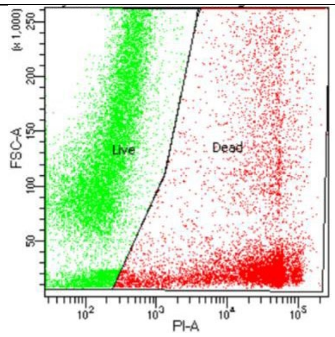	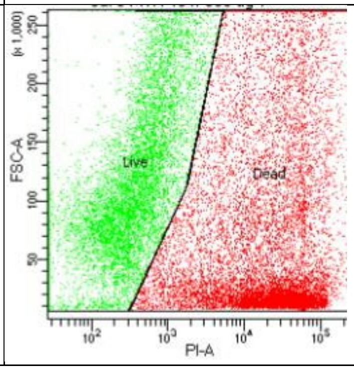	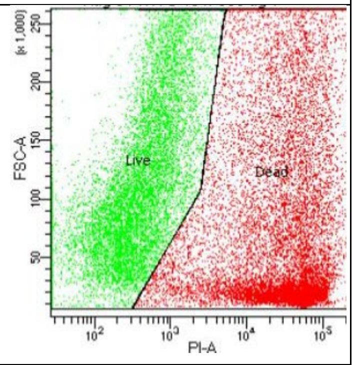

Figure S1. ***(continued)***

Concentration µg/mL	HEO	FGM	AGM
300	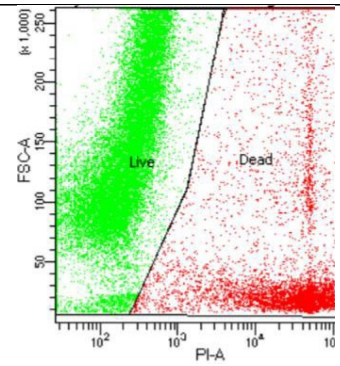	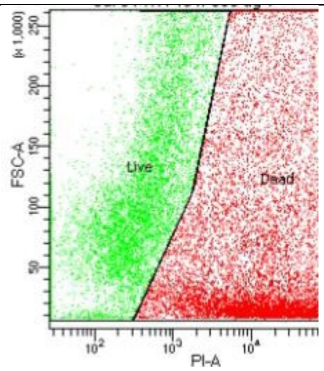	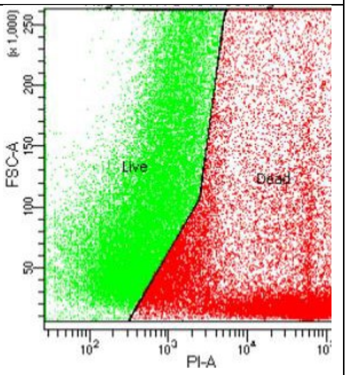
100	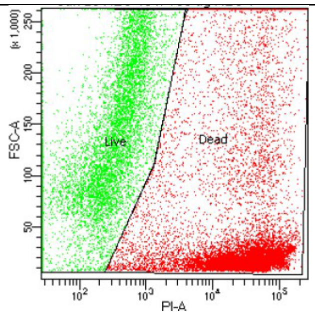	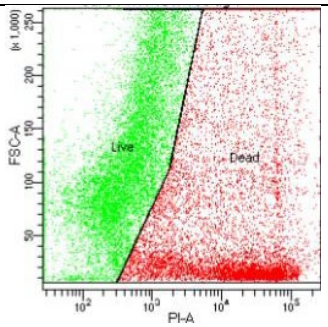	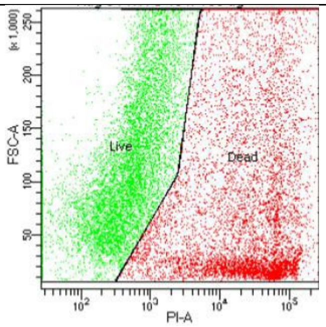
0	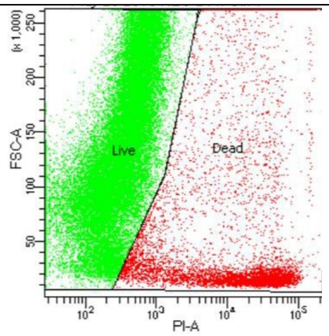	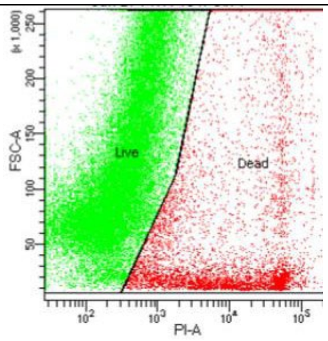	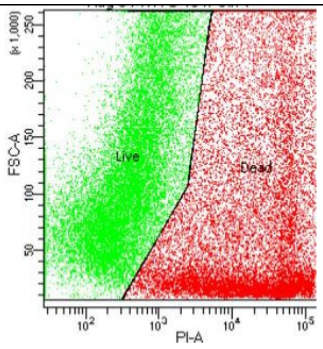

Figure S2. Representative images of HCT-8 cell monolayers after treatment with HEO, FGM, or AGM. All images were captured at 40× magnification; no panel is shown for AGM at 100 µg/mL because this concentration was not tested.

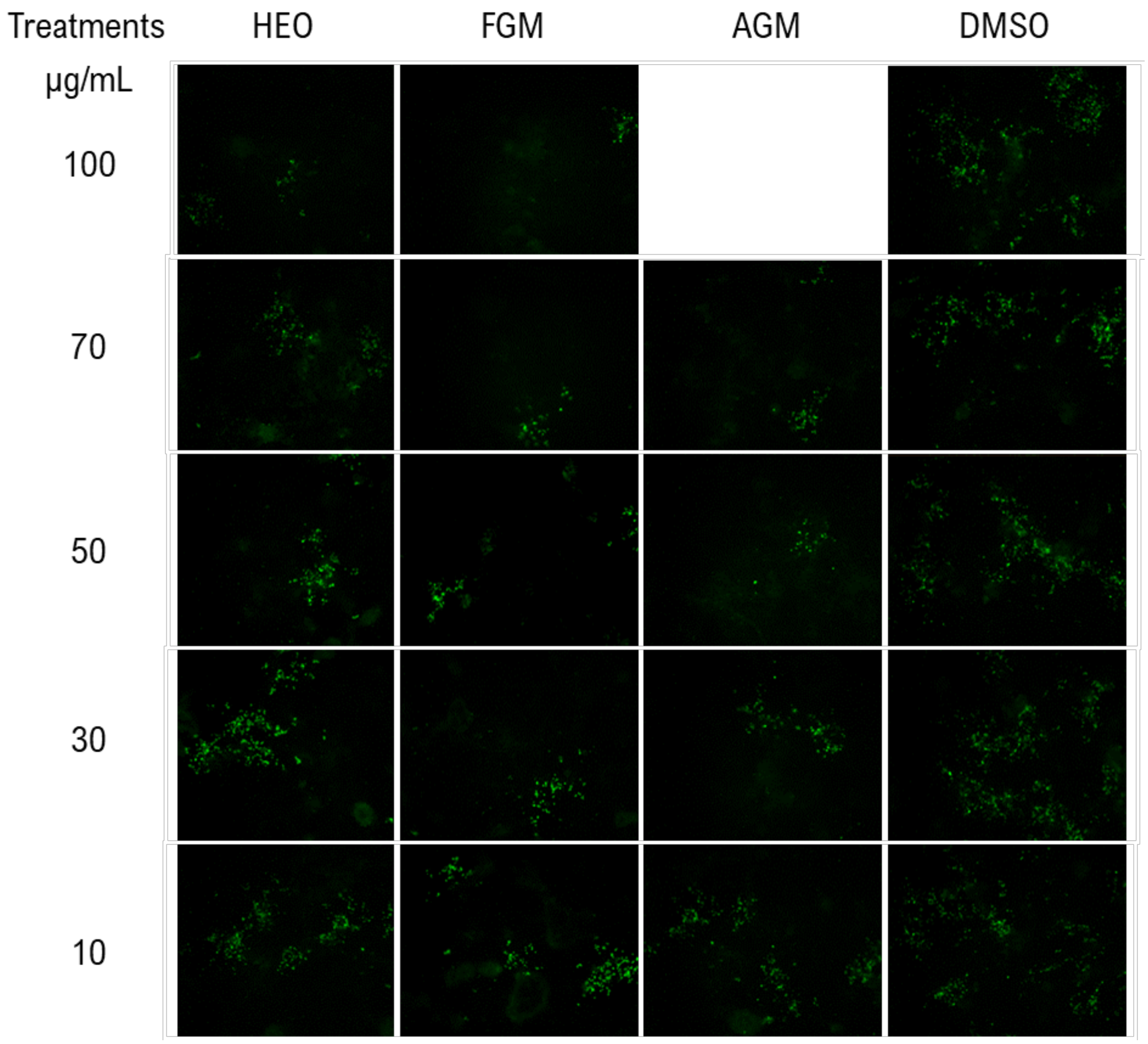

Figure S3. Non-linear sigmoidal dose-response curves for paromomycin in the two experimental modalities. Data points represent means±SD from duplicates across three independent trials. Half-maximal inhibitory concentrations (IC_{50}) were determined using nonlinear regression after normalization in GraphPad Prism v10.4.1.

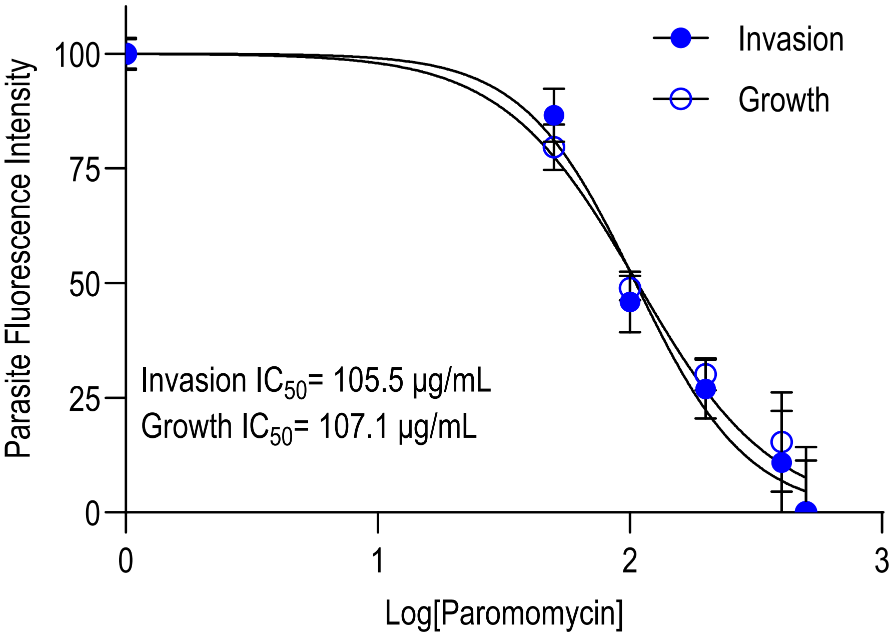

Supplement: Supplementary file 1 [file foods-14-03352-s001.zip › foods-3828325-supplementary.pdf]
